# The Efficacy of CT Temporal Subtraction Images for Fibrodysplasia Ossificans Progressiva

**DOI:** 10.3390/tomography9020062

**Published:** 2023-04-03

**Authors:** Mami Iima, Ryo Sakamoto, Takahide Kakigi, Akira Yamamoto, Bungo Otsuki, Yuji Nakamoto, Junya Toguchida, Shuichi Matsuda

**Affiliations:** 1 Department of Diagnostic Imaging and Nuclear Medicine, Graduate School of Medicine, Kyoto University, 54 Shogoin-Kawaharacho, Sakyo-ku, Kyoto 606-8507, Japan; 2 Institute for Advancement of Clinical and Translational Science (iACT), Kyoto University Hospital, 54 Shogoin-Kawaharacho, Sakyo-ku, Kyoto 606-8507, Japan; 3 Medical Education Center, Kyoto University, Yoshida Konoe-cho, Sakyo-ku, Kyoto 606-8501, Japan; 4 Department of Orthopaedic Surgery, Graduate School of Medicine, Kyoto University, 54 Shogoin-Kawaharacho, Sakyo-ku, Kyoto 606-8507, Japan; 5 Department of Fundamental Cell Technology, Center for iPS Cell Research and Application, Kyoto University, 53 Shogoin-Kawahara-cho, Sakyo-ku, Kyoto 606-8507, Japan

**Keywords:** fibrodysplasia ossificans progressive, bone, computed tomography, image processing, computer-assisted

## Abstract

Purpose: To evaluate the usefulness of CT temporal subtraction (TS) images for detecting emerging or growing ectopic bone lesions in fibrodysplasia ossificans progressiva (FOP). Materials and Methods: Four patients with FOP were retrospectively included in this study. TS images were produced by subtracting previously registered CT images from the current images. Two residents and two board-certified radiologists independently interpreted a pair of current and previous CT images for each subject with or without TS images. Changes in the visibility of the lesion, the usefulness of TS images for lesions with TS images, and the interpreter’s confidence level in their interpretation of each scan were assessed on a semiquantitative 5-point scale (0–4). The Wilcoxon signed-rank test was used to compare the evaluated scores between datasets with and without TS images. Results: The number of growing lesions tended to be larger than that of the emerging lesions in all cases. A higher sensitivity was found in residents and radiologists using TS compared to those not using TS. For all residents and radiologists, the dataset with TS tended to have more false-positive scans than the dataset without TS. All the interpreters recognized TS as useful, and confidence levels when using TS tended to be lower or the same as when not using TS for two residents and one radiologist. Conclusions: TS improved the sensitivity of all interpreters in detecting emerging or growing ectopic bone lesions in patients with FOP. TS could be applied further, including the areas of systematic bone disease.

## 1. Introduction

Fibrodysplasia ossificans progressiva (FOP) is a very rare genetic disorder that causes systemic and progressive ossification in various fibrous tissues such as muscles, tendons, and ligaments throughout the body, often preceded by an episode of painful soft tissue swellings called flare-ups. This leads to a reduced range of motion and ankylosis of limb joints, and reduced mobility and deformity of the trunk [1]. Over the course of their lives, patients develop a second skeleton, resulting in the growing risk of immobility and early death arising from infectious diseases, thoracic insufficiency, and traumatic falls [2].

Plain X-rays, ultrasound, MRI, and CT scans are used at different stages of FOP, and CT is most frequently used for monitoring patients with FOP [3]. MRI is superior to ultrasound for evaluating edema in the early stages of an FOP flare-up, and the early detection of perosseous lesions can also be assessed using MRI [4].

Although the analog method for radiographic assessment of ectopic bone in FOP has been proposed and the strong correlation of ectopic bone lesions with ectopic bone volumes has been demonstrated [5], the evaluation of ectopic bone lesions from radiographs at various anatomic locations cannot be easily performed, which restricts the applicability of this approach in clinical practice.

CT images are more effective than plain radiographs for evaluating ectopic bone lesions of FOP [4], and the sensitivity of whole-body computed tomography (WBCT) is higher than dual-energy X-ray absorptiometry (DXA) for evaluating disease progression in patients with FOP [6]. However, as FOP lesions can arise in any part of the body, evaluating all of the individual lesions in detail takes more time and effort as the number of lesions increases.

Thus, there is a need for a new method that can easily capture image findings and will be useful for the evaluation of disease progression and treatment response in FOP [4].

A temporal subtraction (TS) technique has been developed to subtract a previous image from the current scan using medical images taken at two different points in a time series [7,8,9,10,11,12,13,14]. TS images obtained using an advanced nonrigid image registration algorithm, termed large deformation diffeomorphic metric mapping (LDDMM), have been found useful for detecting various lesions such as lung nodules [9], bone metastases [12,14], and cerebral infarctions [13]. The TS image technique is considered beneficial for analyzing focal temporal changes, especially in ectopic ossification diseases such as FOP, which require a detailed evaluation of lesions occurring in any part of the whole body. Thus, the purpose of this study was to evaluate the usefulness of TS images in the detection of emerging or growing ectopic bone lesions in patients with FOP.

## 2. Materials and Methods

### 2.1. Study Population

This retrospective study was approved by our institutional review board and included four patients (16, 18, 25, and 55 years old, three male and one female) diagnosed with FOP. CT scans were performed between 1 March 2016 and 31 December 2019. One patient’s images were selected for interpreters to practice evaluating TS images and were thus not included in the analysis. Out of the remaining three patients, two were scanned three times and one was scanned four times using CT (Aquilion PRIME, Aquilion ONE Vision Edition, Canon Medical Systems, Otawara, Japan). The CT images were reconstructed with a slice thickness of 1 mm using a soft-tissue kernel (FC13). The interval of the scans was 201 ± 59 days (mean and standard deviation).

### 2.2. Generation of TS Images

The TS images were generated using a dedicated workstation: Vitrea SURE Subtraction Time Lapse Ortho (Canon Medical Systems, Otawara, Japan). In brief, following the nonrigid registration of the previous and current CT images using LDDMM, TS images were produced by subtracting the previously registered CT images from the current CT images in each interval. Thus, seven datasets of TS images (two patients for 2 intervals and one patient for 3 intervals) were generated and evaluated by interpreters (Table 1).

The detailed process for obtaining TS images is described in a previous report [14].

### 2.3. Searching Ectopic Bone Lesions

Two residents (M.K. and E.N.) with 1–2 months’ experience interpreting CT images and two board-certified radiologists (M.I. and T.K.) with more than ten years of experience independently interpreted a pair of current and previous CT images for each subject with and without TS images in the axial orientation. All interpreters were familiarized with TS images by reading two datasets of TS images from one patient prior to the study evaluation; the practice readings were not included in the analysis. The interpreters were allowed to change the window level and window width. They were informed of the patient’s age and sex and that the patient had FOP, and they were blinded to all other clinical data.

Image interpretation was conducted twice for each image dataset, with and without TS images. The order of the datasets was randomized. One resident and one radiologist interpreted first without TS images and then with TS images, and vice versa for the other resident and radiologist. An interval of more than 30 days was set between the two reading sessions to minimize the memory effect.

They were asked to identify newly emerging or growing ectopic bone lesions in each interval. Ectopic bone lesions were defined as hyper intense lesions (over 200 H.U., approximately) outside or adjacent to normal skeletal bone, such as in the subcutaneous regions, muscles, and skeletal joints; the evaluation range was from the first thoracic spine level to that of ischial tuberosity. Emerging lesions were those that did not exist in the previous image, but appeared in the current image; growing lesions were those that existed in the previous image and were enlarged in the current image.

The other board-certified radiologist (R.S.), with more than ten years of experience, reviewed the CT images to evaluate the emerging or growing ectopic bone lesions between the two serial scans, and these evaluations were defined as the reference standards. R.S. then judged all of the lesions detected by the interpreters as identical or not identical to the reference standards.

### 2.4. Evaluation of TS Images

The following three surveys were conducted, and evaluation items were scored on a 5-point scale:

- Survey 1: The visibility of lesion changes on a per-lesion basis (0, difficult to see; 1, slightly difficult to see; 2, neither hard nor easy to see; 3, slightly easy to see; 4, easy to see).
- Survey 2: The usefulness of TS images to identify lesions on a per-lesion basis (0, useless; 1, not very useful; 2, somewhat useful; 3, very useful; 4, extremely useful).
- Survey 3: The confidence level of the interpreter in their interpretation of each scan (0, very low; 1, low; 2, moderate; 3, high; 4, very high).

The reading time of each session was also recorded.

### 2.5. Statistical Analysis

Lesion-based sensitivity and false-positive rates per scan were estimated.

The Wilcoxon signed-rank test was used to assess differences in the scores of survey 1 and survey 3 between the datasets with and without TS images. The statistical analysis was performed using MedCalc version 20.211 (MedCalc, Mariakerke, Belgium).

## 3. Results

The number of emerging and growing lesions as the reference standard is described in Table 1. The number of growing lesions tended to be greater than the number of emerging lesions in all cases.

TS images were successfully generated in all cases. The processing time used in this study was approximately 30 min per case.

The diagnostic performance in detecting newly ectopic bone lesions is shown in Table 2. The detection sensitivity of both residents and radiologists tended to be greater when using TS compared to when not using TS. The false-positive rates per scan tended to increase when using TS for both residents and radiologists.

Table 3 shows the scores for the visibility of new lesions (survey 1), the usefulness of TS images for identifying lesions (survey 2), the confidence levels of the interpreters in their interpretation for each scan (survey 3), and the reading time for each interpreter. The scores of survey 1 were comparable for all interpreters. In survey 2, all interpreters recognized TS as useful, and the radiologists tended to find TS more useful than the residents. The confidence levels for most interpreters tended to be the same or decreased when using TS compared to when not using TS, except for the confidence level of radiologist A, which was significantly higher when using TS.

The reading times decreased when using TS compared to when not using TS for all interpreters, except for resident A (Table 3).

### Representative Cases

**Figure 1:**
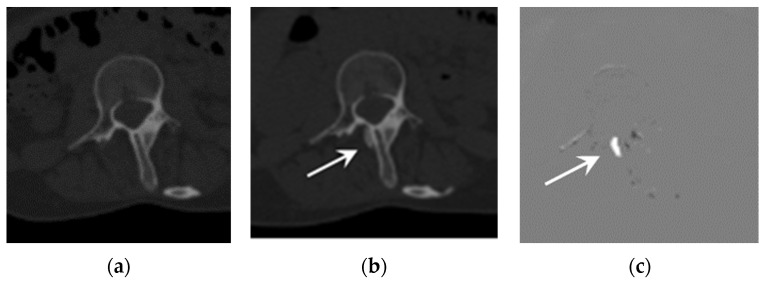
Example of an emerging lesion. CT images of a 25-year-old FOP patient: (**a**) previous CT image, (**b**) current CT image, and (**c**) TS (subtraction) image. The emerging lesion adjacent to the thoracic spine (arrow) is difficult to detect on the current CT image, but is clearly visible in the TS image.

**Figure 2:**
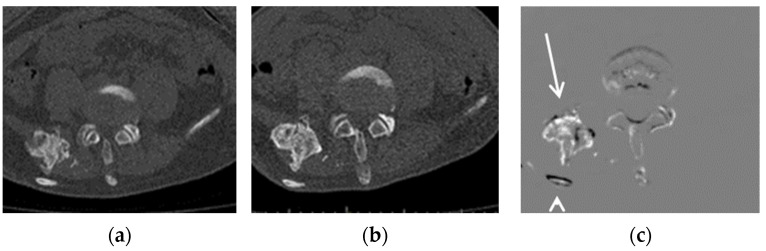
Example of a growing lesion. CT images of a 16-year-old FOP patient: (**a**) previous CT image, (**b**) current CT image, and (**c**) TS (subtraction) image. The growing lesion near the ilium (arrow) is difficult to detect in the current CT image, but is clearly visible in the TS image. The dorsal rib of this lesion (arrowhead) is shown due to misregistration.

Representative cases are shown in Figure 1 and Figure 2. Both cases showed better detectability of emerging or growing ossifications when using TS compared to when not using TS.

## 4. Discussion

This study investigated the utility of TS imaging by evaluating observers’ performance in detecting emerging or growing ectopic bone lesions in FOP patients. The accurate detection of new ectopic bone lesions during the follow-up of FOP patients is considered important in routine diagnostic imaging, which requires professional and substantial efforts by radiologists during routine CT scans, and would be challenging for orthopedists or pediatricians who consult with FOP patients in clinical practice.

Sensitivity when using CT images with TS in detecting emerging or growing ectopic bone lesions was found to be higher than when using CT images without TS, which was in agreement with the previous study investigating the TS detectability of bone metastases [15]. These results indicate the usefulness of TS in the detection of emerging or growing ectopic bone lesions, which is sometimes challenging with conventional CT images, as these lesions can develop in any part of the skeleton [16] and are uncommon for radiologists as well as residents. In addition, there is no additional acquisition time or radiation exposure necessary for generating TS images, as TS images are calculated from CT images acquired in routine clinical practice. The processing time was comparable to that reported in a previous study [14,15], and drastically shortened compared to the initial investigation [12] due to the improvements in the TS processing software. We expect that, with a shorter processing time, TS can be made more readily available in clinical settings.

Sensitivity when using TS to detect new ectopic lesions was 30.2–51.2%, which was inferior to the results reported for a previous investigation (54%) that evaluated the diagnostic performance value of TS in detecting bone metastases. Sensitivity was also variable, perhaps due to the rarity of FOP patients and thus the lack of experience in reading such images. However, a slightly inferior sensitivity in the detection of bone lesions in FOP patients compared to bone metastasis in cancer patients would still be of value for both patients and radiologists, providing more effective and efficient detection of issues that are challenging to detect due to the nonspecific location of potential lesions and the rarity of the disease.

In addition to the improvement in sensitivity, the number of false positives per case when using TS tended to be higher than when not using TS for three out of the four interpreters. This was partially due to the misregistrations of TS in some cases (Figure 2 and [12]). It is well known that the image quality of TS images significantly affect diagnostic performance [17], and a further improvement in the image registration algorithm would be desirable for more accurate diagnosis.

All the interpreters found the TS images useful for detecting new bone lesions (3–4 scores); however, the visibility of FOP lesions showed no significant difference, whether using TS or not. Once the interpreters had detected the new bone lesions, there seems to have been little value added by TS in their visibility, as they were calcified lesions with high contrast in CT images.

The level of confidence in their interpretation of each scan was variable among interpreters, except for the significantly higher confidence level of radiologist A when using TS compared to when not using TS. Ectopic bone lesions, unlike bone metastases, often occur adjacent to normal bones [3,18], such as continuous sclerosis protruding from the bone or isolated periosteal sclerosis, as opposed to a lesion within the normal bone. As the error signals arising from misregistration in TS appear at the edge of bones [12], three interpreters might have had difficulty in distinguishing true ectopic bone lesions from misregistration errors when reading TS.

The reading time was significantly shortened when using TS for each interpreter except for resident A, which indicates the ability of TS to provide an efficient diagnostic enhancement in the detection of new bone lesions. The significantly longer TS image reading time for one resident might have been due to the complexity of the evaluation task.

Our study suggests that bone TS images have the potential to be applied in other areas, including the detection of systemic bone diseases, such as osteomalacia, ankylosing spondylitis, or diffuse idiopathic skeletal hyperostosis. As comparison of images at two time points is fundamental to evaluating lesion progression, the TS technique is expected to be used in a wide range of diseases.

There are several limitations in this study. First, this was a retrospective single-center study with a small number of patients, because FOP is a rare disease.

A study with a larger population and longer duration will need to be conducted in the near future to validate these results. Moreover, the prediction of ectopic bone emergence could be investigated, as CT analysis has been found to be useful for the prediction of bone lesions’ emergence, such as metastasis. Changes in bone mineral density obtained from CT images might also be potentially sensitive to fracture-related bone changes [11].

Second, there was a small number of interpreters, as this was a preliminary study to investigate whether TS images can be applied to follow-up evaluation of FOP lesions. Third, only pairs of thin-slice (1 mm) CT images were evaluated, whereas pairs of thin-slice CT images are not always available in clinical settings. Fourth, the generation of TS images requires both current and previous images. This method might not be useful for the initial evaluation, however, it is worth considering in the follow-up of lesions in the long term.

In conclusion, this evaluation of the use of TS in detecting emerging or growing ectopic bone lesions in FOP patients showed a higher sensitivity among residents and radiologists. However, there were more false-positive scans in datasets evaluated using TS compared to those without TS. TS might provide better diagnostic performance in follow-ups of FOP patients.

## Figures and Tables

**Table 1:** The number of emerging and growing lesions for each case and their intervals.

	Interval 1		Interval 2		Interval 3		
	Emerging	Growing	Emerging	Growing	Emerging	Growing	
Case A	0	6	0	5	n/a	n/a	
Case B	0	2	0	3	n/a	n/a	
Case C	4	4	4	6	4	5	
Total	4	12	4	14	4	5	43

Cases A and B had two scans each, and Case C had three scans at follow-up.

**Table 2:** The diagnostic performance in detecting newly ectopic bone lesions without and with TS.

		Resident A	Resident B	Radiologist A	Radiologist B
Sensitivity (%)	Without TS	25.6	14.0	16.3	20.9
	With TS	51.2	30.2	41.9	46.5
Number of false-positive cases	Without TS	0	0	2	0
	With TS	7	3	1	1

**Table 3:** The scores for each survey and the reading times.

		Resident A	Resident B	Radiologist A	Radiologist B
Survey 1	Without TS	2 (0–4)	2 (0–3)	2 (0–4)	4 (0–4)
	With TS	1.5 (0–4)	2 (1–3)	2 (1–4)	4 (3–4)
Survey 2		3 (0–4)	3 (1–4)	4 (0–4)	4 (1–4)
Survey 3	Without TS	3 (1–3)	0 (0–2)	2 (1–3)	4 (4–4)
	With TS	2 (2–4)	0 (0–2)	3 (3–4) *	3 (1–4)
Reading time	Without TS	718 ± 266	1543 ± 477	814 ± 162	429 ± 160
	With TS	1920 ± 876	891 ± 258	780 ± 211	437 ± 161

Survey 1: Visibility of new lesions (0, difficult to see; 1, slightly difficult to see; 2, neither hard nor easy to see; 3, slightly easy to see; 4, easy to see). Survey 2: Usefulness of TS images for identifying lesions (0, useless; 1, not very useful; 2, somewhat useful; 3, very useful; 4, extremely useful). Survey 3: Confidence level of the interpreter in their interpretation of each scan (0, very low; 1, low; 2, moderate; 3, high; 4, very high) on a 5-point scale. Median values with ranges are shown for each survey. Reading times are demonstrated as means with standard deviations. * Significant difference (*p* = 0.02).

## Data Availability

Not applicable.
